# Targeting ZDHHC21/FASN axis for the treatment of diffuse large B-cell lymphoma

**DOI:** 10.1038/s41375-023-02130-5

**Published:** 2024-01-09

**Authors:** Bangdong Liu, Xianlan Zhao, Shihao Zhang, Qiong Li, Xinlei Li, Dezhi Huang, Jing Xia, Naya Ma, Yishuo Duan, Xi Zhang, Jun Rao

**Affiliations:** 1 Medical Center of Hematology, Xinqiao Hospital, Army Medical University, Chongqing, China; 2 State Key Laboratory of Trauma and Chemical Poisoning, Chongqing Key Laboratory of Hematology and Microenvironment, Jinfeng Laboratory, Chongqing, China; 3 Department of Basic Medicine, Zhongshan School of Medicine, Sun Yat-sen University, Guangzhou, China

**Keywords:** B-cell lymphoma, Cancer metabolism, Cell signalling, Targeted therapies

## Abstract

S-palmitoylation is essential for cancer development via regulating protein stability, function and subcellular location, yet the roles S-palmitoylation plays in diffuse large B-cell lymphoma (DLBCL) progression remain enigmatic. In this study, we uncovered a novel function of the palmitoyltransferase ZDHHC21 as a tumor suppressor in DLBCL and identified ZDHHC21 as a key regulator of fatty acid synthetase (FASN) S-palmitoylation for the first time. Specifically, ZDHHC21 was downregulated in DLBCL, and its expression level was associated with the clinical prognosis of patients with DLBCL. In vitro and in vivo experiments suggested that ZDHHC21 suppressed DLBCL cell proliferation. Mechanistically, ZDHHC21 interacted with FASN and mediated its palmitoylation at Cys1317, resulting in a decrease in FASN protein stability and fatty acid synthesis, consequently leading to the inhibition of DLBCL cell growth. Of note, an FDA-approved small-molecule compound lanatoside C interacted with ZDHHC21, increased ZDHHC21 protein stability and decreased FASN expression, which contributed to the suppression of DLBCL growth in vitro and in vivo. Our results demonstrate that ZDHHC21 strongly represses DLBCL cell proliferation by mediating FASN palmitoylation, and suggest that targeting ZDHHC21/FASN axis is a potential therapeutic strategy against DLBCL.

## Introduction

Diffuse large B-cell lymphoma (DLBCL) is the most common hematologic malignancy [1]. Although almost half of patients can achieve long-term remission with standard rituximab, cyclophosphamide, doxorubicin, vincristine, and prednisone (R-CHOP) immunochemotherapy, the majority of relapsed patients succumb to DLBCL [2, 3]. Therefore, it is necessary to explore novel therapeutic strategies for patients with DLBCL.

DLBCL cells are characterized by upregulation of fatty acid synthesis and are highly addictive to lipids for cell growth, regardless of their cell of origin [4–7]. Furthermore, fatty acid synthase (FASN), a key multienzyme complex responsible for de novo lipogenesis, is highly upregulated in DLBCL, but the mechanism underlying FASN overexpression remains unclear [6]. Previous studies have found that inhibiting FASN activity resulted in a robust decline in tumor growth and dramatically induced apoptosis in DLBCL [5, 8]. Nevertheless, current inhibitors targeting FASN have restricted clinical applications because of their certain pharmacological limitations [9]. Therefore, exploring the molecular mechanism underlying the significantly enhanced expression of FASN may contribute to more effective therapeutic strategies for DLBCL.

Protein S-palmitoylation is a reversible post-translational modification that refers to the thioesterification of a 16-carbon fatty acid (palmitate) covalently bound to internal cysteine residues [10]. To date, emerging studies have revealed that protein palmitoylation plays a crucial role in various diseases, especially cancers, by altering the subcellular localization, protein stability and function of important oncogenes and tumor suppressors [10–13]. For example, glucose transporter member 1 (GLUT1) S-palmitoylation is required for maintaining its plasma membrane localization and promotes glioblastoma tumorigenesis [14]. Another study found that S-palmitoylation stabilizes PD-L1 by blocking its ubiquitination, consequently inhibiting PD-L1 degradation and contributing to the immune escape of breast cancer cells [15].

In mammalian cells, S-palmitoylation is catalyzed by 23 members of the Asp-His-His-Cys (DHHC)-family of palmitoyl S-acyltransferases, and palmitate removal is mediated by serine hydrolases, including acyl-protein thioesterases [11, 16, 17]. A previous study found that ZDHHC21 is required for endogenous androgen receptor palmitoylation, membrane localization, and signal transduction in breast cancer cells [18]. Moreover, a recent study reported that ZDHHC21 could regulate oxidative phosphorylation by mediating adenylate kinase 2 (AK2) S-palmitoylation and induce differentiation block and stemness in acute myeloid leukemia [19]. Thus, targeting protein S-palmitoylation or enzymes involved in S-palmitoylation dynamics has valuable clinical potential and can represent candidate therapies for certain cancers. However, the roles S-palmitoylation or the responsible enzyme plays in lymphomagenesis and cancer development remain enigmatic.

In the present study, we demonstrated for the first time that the palmitoyltransferase ZDHHC21 interacted with and palmitoylated FASN at Cys1317, which led to a decrease in FASN protein stability and fatty acid synthesis and consequently resulted in the inhibition of DLBCL cell proliferation. Of note, an FDA-approved small-molecule compound, lanatoside C, was found to combine with ZDHHC21 and suppress DLBCL cell growth in vitro and in vivo by regulating ZDHHC21/FASN axis and fatty acid synthesis. Taken together, our results not only reveal a novel biological function of ZDHHC21 as a tumor suppressor in DLBCL and identify it as a key regulator for FASN palmitoylation for the first time but also establish targeting ZDHHC21/FASN axis as a promising therapeutic strategy for patients with DLBCL.

## Materials and methods

### Cell culture

Human DLBCL cell lines, including SU-DHL-2, SU-DHL-4, SU-DHL-6, Toledo and Pfeiffer, were purchased from American Type Culture Collection (ATCC; Manassas, VA) and maintained in RPMI-1640 supplemented with 10% FBS. OCI-LY1 and OCI-LY3 cells were maintained in IMDM supplemented with 10% FBS. HEK293T cells were maintained in DMEM supplemented with 10% FBS. All cell lines were authenticated using short tandem repeat (STR) DNA profiling and verified to be mycoplasma-free.

### Patients and specimens

Tissue microarrays of human DLBCL and normal tissues (HLymB085PT01) were purchased from Shanghai Outdo Biotech Company (Shanghai, China). For the use of these clinical materials for research purposes, prior patients’ consents and approval from the Institutional Research Ethics Committee were acquired.

### RT-PCR

Reverse transcription and real-time quantitative PCR using SYBR PCR Master Mix (Vazyme Biotech, Nanjing, China) were performed with specific RT-PCR primers. The primers used for the amplification of indicated genes were listed in Supplementary Table 1.

### Western blot analysis

The primary antibodies used in this study were anti-ZDHHC21 (Novus, Cat# NBP1-57049), anti-Flag (CST, Cat# 8146), anti-HA (CST, Cat# 3724), anti-FASN (Santa Cruz, Cat# sc-48357), anti-FASN (CST, Cat# 3180), anti-biotin (CST, Cat# 7075), anti-α-tubulin (Proteintech, Cat# 11224-1-AP) and anti-Myc (CST, Cat# 2278).

### Co-immunoprecipitation and mass spectrometry analysis

HEK293T cells were transfected with vector or ZDHHC21-Flag plasmid for 48 h and then lysed with 1 ml lysis buffer [20 mM Tris-HCl (pH 7.4), 300 mM NaCl, 1% Triton-X100, 1 mM EDTA and 1 mM PMSF]. Anti-Flag magnetic beads were washed with 1 ml lysis buffer containing 150 mM NaCl three times and incubated with cell lysate overnight at 4°C. Then, SDS-PAGE and silver staining were performed, and whole bands were excised for MS analysis by Applied Protein Technology (Shanghai, China).

### CCK8 assay

To determine whether ZDHHC21 suppresses the growth of DLBCL cells in vitro, 5 × 10^3^ cells in which ZDHHC21 was overexpressed or depleted were resuspended in 180 μl culture medium and inoculated in 96-well plates. Next, 20 μl CCK8 reagent was added, and the absorbance at 450 nm was measured after 2 h. For the lanatoside C treatment study, 8 × 10^3^ OCI-LY1 and SU-DHL-2 cells were inoculated in 96-well plates, and the absorbance at 450 nm was measured.

### Acyl-biotinyl exchange (ABE) assay

The ABE assay was performed as previously described with slight modifications [20]. In brief, DLBCL cells were harvested and washed with 1 ml PBS twice and then lysed in 1 ml lysis buffer [100 mM Tris-HCl (pH 7.2), 150 mM NaCl, 5 mM EDTA, 2.5% SDS, 5 mM PMSF]. The cell lysates were incubated with 10 mM N-ethylmaleimide (NEM) at 4 °C overnight to cap the free cysteine residues and then purified by sequentially adding methanol, chloroform and distilled water in a ratio of 4:1.5:3 relative to sample volumes. The protein pellets were resuspended in 1.2 ml resuspension buffer [100 mM Tris-HCl (pH 7.2), 150 mM NaCl, 5 mM EDTA, 2.5% SDS, 8 M urea], purified two more times to thoroughly remove the excess NEM and then incubated with 0.5 M hydroxylamine (HAM) for 1 h at room temperature to cleave palmitoylation thioester bonds. Then, the mixture was incubated with 0.1 mM biotin-HPDP for 1 h at room temperature. The samples were purified by methanol-chloroform-water precipitation and then resuspended in resuspension buffer, boiled at 100 °C for 10 min, subjected to SDS-PAGE, and analyzed by immunoblotting.

### Metabolic labeling and click reaction

Metabolic labeling and click reaction assays were performed according to the published procedure with slight modifications [14, 21]. In brief, cells were labeled with alkynyl probes for 12 h, harvested, washed with 1 ml ice-cold DPBS and lysed with 1 ml lysis buffer [50 mM Tris-HCl (pH 7.4), 150 mM NaCl, 0.1% Triton X-100, 0.1% SDS, 5 mM PMSF] supplemented with 50 mM NEM. The next steps were performed as described in the Click Chemistry Tools web server.

### Free fatty acid assay

Content of free fatty acid was detected using Free Aliphatic Acid Assay Kit (Bioss, Cat # AK230). In brief, DLBCL cells were lysed and the free fatty acid combined with copper ions to form copper salts of fatty acid which dissolved in chloroform and was later measured by spectrophotometric method at 550 nm. The content of free fatty acid was calculated using copper ion concentration, and the free fatty acid content of ZDHHC21 overexpressed or silenced cells was compared with that of vector cells.

### FASN activity assay

FASN enzyme activity was detected using Fatty Acid Synthetase Activity Assay Kit (Abbkine, Cat # KTB2240). 5×10^6^ DLBCL cells were collected and lysed with 1 mL Extraction Buffer. Then, 20 μL sample, 16 μL NADPH, 4 μL Acetyl CoA, 8 μL Malonyl CoA and 152 μL Assay Buffer were added to a 96-well plate, and the absorbance value at 340 nm was measured at 37 °C. The 10 s and 70 s absorbance values were recorded to calculate FASN enzyme activity.

### Molecular docking

Molecular docking calculations were conducted using the Dock6 program in Yinfo Cloud Computing Platform. The 3D structure of small-molecule library of FDA-approved drugs was constructed with energy minimization in MMFF94 force field. The 3D structure of ZDHHC21 protein (PDB code: AF_AFQ8IVQ6F1) was downloaded from the RCSB Protein Data Bank (http://www.rcsb.org/).

### Tumor xenograft assay

All experimental procedures were approved by the Institutional Animal Care and Use Committee of Army Medical University. For xenograft experiments, 6-week-old female BALB/c nude mice were purchased from Beijing Vital River Laboratory Animal Technology (Beijing, China) and maintained in microisolator cages.

To evaluate whether ZDHHC21 suppresses the growth of DLBCL cells in vivo, 1 × 10^7^ cells in which ZDHHC21 was overexpressed or - depleted were resuspended in 100 μl PBS and subcutaneously injected into BALB/c nude mice. Tumor volumes were measured every two days, the BALB/c nude mice were sacrificed after nearly four weeks, and xenograft tumors were removed and weighed.

For the lanatoside C treatment study, 1 × 10^7^ SU-DHL-6 cells were subcutaneously injected into BALB/c nude mice. When tumor volumes reached nearly 100 mm^3^, vehicle or lanatoside C (6 mg/kg) was administered intraperitoneally every two days, and the tumor volumes and mouse body weights were measured every two days. Then, the mice were sacrificed, and xenograft tumors were obtained. At least five nude mice per group were used to ensure the adequate power and each mouse with different weight was randomly allocated. Measurements of tumor volumes and weights were not performed in a blinded manner.

### Bioinformatics and statistical analysis

DLBCL-specific ZDHHC21 RNA expression data were downloaded from the GEO datasets (https://www.ncbi.nlm.nih.gov/). Some samples of the GSE10846 and GSE53786 cohorts used for survival analysis were deleted due to loss of patients’ clinical information and the log-rank test was used to analyze overall survival in patients with DLBCL. Comparisons between two groups were analyzed using the unpaired, two-tailed Student’s t test. For pairwise multiple comparisons, one-way ANOVA followed by Dunnett’s multiple comparison test was used as appropriate. The sample size was determined by power analysis to achieve a minimum effect size of 0.5 with *p* < 0.05, and all sample sizes were appropriate for the assumption of normal distribution. Variance within each group of data was estimated and was similar between compared groups. Data analysis was performed by two independent investigators who were blinded to the sample groups. All error bars represent the mean ± SD derived from three independent experiments. *P* values < 0.05 were considered statistically significant.

## Results

### ZDHHC21 expression is downregulated in DLBCL and correlates with poor prognosis

**Fig. 1:**
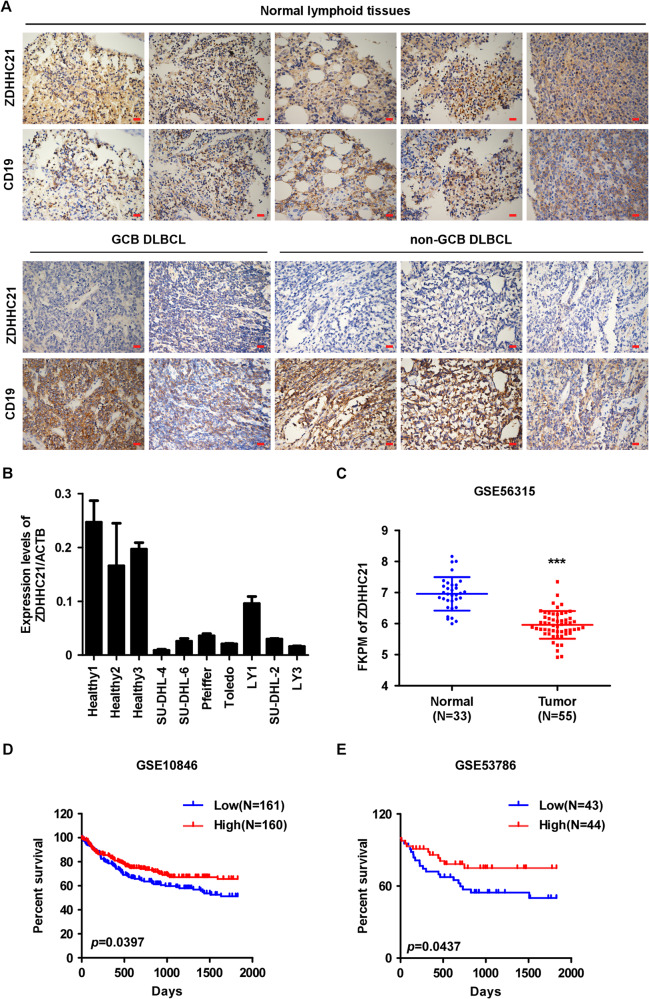
ZDHHC21 expression is downregulated in DLBCL with prognostic value. **A** The expression level of ZDHHC21 in normal lymphoid tissues and DLBCL tumor tissues with CD19 as a B-cell marker, as evaluated by IHC assay (magnification ×400, scale bar, 20 μm). **B** The expression of ZDHHC21 is downregulated in DLBCL cell lines compared with B lymphocytes derived from healthy volunteers using qRT-PCR analysis (each bar represents the mean ± SD derived from three independent experiments). **C** Analyses of ZDHHC21 expression in GSE56315 DLBCL cohorts (two-tailed Student’s t test. ***p < 0.001). **D**, **E** Kaplan–Meier survival analysis of overall survival in the GSE10846 (**D**) and GSE53786 (**E**) DLBCL cohorts based on ZDHHC21 expression, with ZDHHC21 median expression level as the cutoff value to define “high” and “low”.

We began our study by examining the expression level of ZDHHC21 in archived tissue specimens using immunohistochemical (IHC) staining. Serial sections of human DLBCL and normal lymphoid tissues were used to detect ZDHHC21 and CD19 expression, which acted as a B-cell marker. As shown in Fig. 1A, ZDHHC21 expression was significantly downregulated in both germinal center B-cell like (GCB) and non-GCB DLBCL tissues, compared with that in CD19 positive cells of normal lymphoid tissues. Consistently, the expression level of ZDHHC21 in 7 DLBCL cell lines was evidently decreased as exhibited by RT-PCR assay (Fig. 1B). Next, we employed GEO datasets to identify the expression level of ZDHHC21 mRNA in patients with DLBCL. As shown in Fig. 1C, ZDHHC21 expression was markedly declined in DLBCL tissues compared with that in B cells from normal human tonsils. To explore the circumstances of low ZDHHC21 expression in DLBCL, we firstly performed ZDHHC21 copy number variation analysis of 48 DLBCL cases in The Cancer Genome Atlas (TCGA) dataset and found that 3 out of 48 cases (6.25%) showed heterozygous deletion in ZDHHC21 locus. Next, we assessed the mutation status of ZDHHC21 in cBioPortal for Cancer Genomics dataset and the results suggested that only 1 out of 135 cases (0.74%) showed mutation in ZDHHC21 locus. Moreover, methylation status of ZDHHC21 in TCGA DLBCL cohort was also analyzed. There are nine methylation specific probes targeting ZDHHC21 locus and the correlation between them and ZDHHC21 expression was analyzed. As shown in Supplementary Fig. 1A, B, the methylation level of probe cg01785060 and cg00775695 significantly negatively correlated with ZDHHC21 expression level, suggesting that their methylation level might regulate the expression of ZDHHC21. Whereafter, EWAS DNA methylation database was employed and we found that there existed no significant difference between the methylation level of DLBCL patients and healthy lymph node control. Besides, transcriptional activation or repression mediated by transcription factor is also an important way to regulate gene expression. DLBCL is characterized by activation of NF-κB, JAK/STAT3 and other pathways [3, 22]. To further investigate whether these transcription factors could regulate ZDHHC21 expression in DLBCL, we searched the promoter sequence of ZDHHC21 using JASPAR database and the results showed that there existed potential binding sites of NFKB1, NFKB2 and STAT3 (Supplementary Fig. 1C), and we further found that NFKB1 and NFKB2 expression significantly negatively correlated with ZDHHC21 expression in ABC subtype DLBCL patients but not in GCB subtype according to GSE53786 cohort. However, STAT3 expression and ZDHHC21 expression was significantly negatively correlated in GCB subtype DLBCL patients but not in ABC subtype (Supplementary Fig. 1D). The above results suggested that NFKB1, NFKB2 and STAT3 might repress ZDHHC21 transcription by binding its promoter. Taken together, deletion in ZDHHC21 locus and transcriptional regulation mediated by transcription factor might contribute to the low expression of ZDHHC21 in DLBCL.

Meanwhile, Kaplan–Meier survival analysis showed that low expression of ZDHHC21 was associated with shorter overall survival times in patients diagnosed with DLBCL (Fig. 1D, E). Next, we combined the International Prognostic Index (IPI) with ZDHHC21 expression (with ZDHHC21 low expression defined as 1 point) and established the IPI-21 system, allowing greater discrimination among highest-risk and lowest-risk patients (Supplementary Fig. 2A–D). Taken together, these data indicated a significant downregulation of ZDHHC21 with prognostic value in DLBCL, warranting further investigation about whether downregulated ZDHHC21 plays a role in the progression of DLBCL.

### ZDHHC21 suppresses DLBCL cell proliferation in vitro

**Fig. 2:**
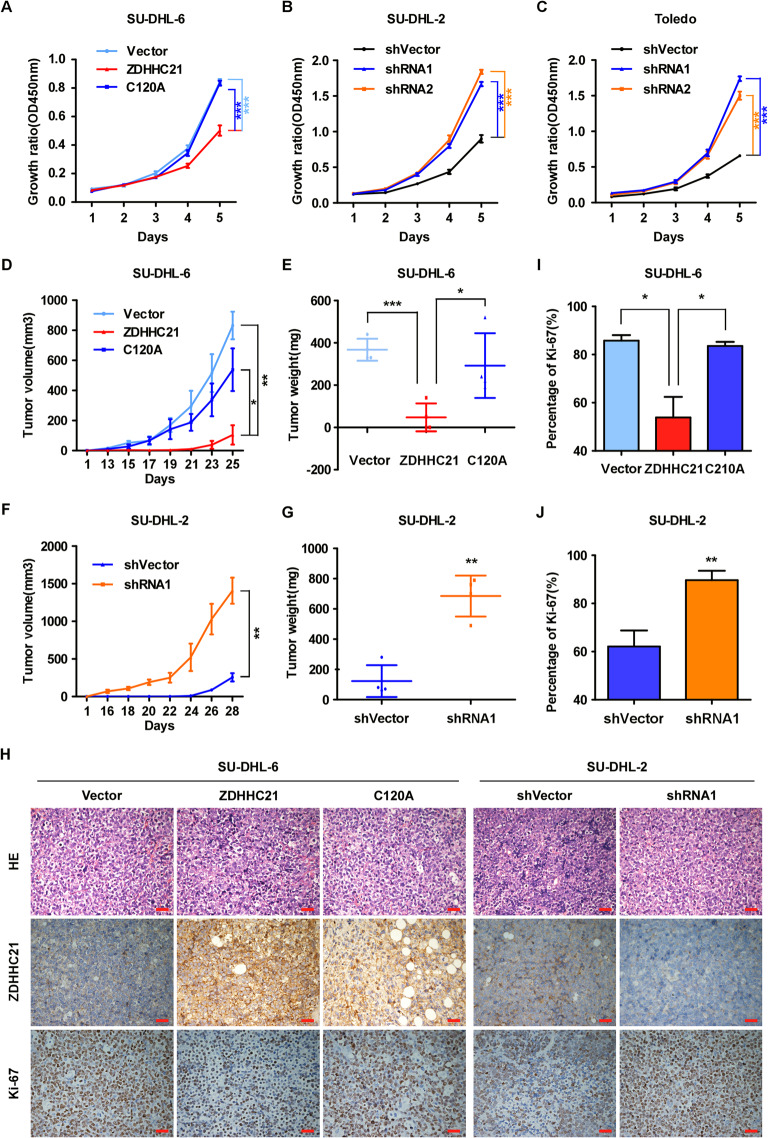
ZDHHC21 inhibits DLBCL tumor growth in vitro and in vivo. **A**–**C** The growth rate of cells in which ZDHHC21 is stably overexpressed (**A**) and depleted (**B**, **C**) was examined with CCK8 assay (each bar represents the mean ± SD derived from three independent experiments, one-way ANOVA followed by Dunnett’s multiple comparison test, ***p < 0.001). **D**, **E** The control-vector, ZDHHC21-WT and ZDHHC21-C120A cells were subcutaneously inoculated into nude mice. We measured tumor volume every two days from day 13 to day 25, and the mice were sacrificed on day 25. Tumor growth curve (**D**) and tumor weight at the end of the experiments (**E**) are shown (each bar represents the mean ± SD derived from four independent experiments, one-way ANOVA followed by Dunnett’s multiple comparison test, *p < 0.05, **p < 0.01, **p < 0.001). **F**, **G** The control-vector and ZDHHC21-depleted cells were subcutaneously inoculated into nude mice. Tumor volume was measured once every two days from day 16 to day 28. Tumor growth curve (**F**) and tumor weight at day 28 (**G**) are shown (each bar represents the mean ± SD derived from four independent experiments, two-tailed Student’s t test, **p < 0.01). **H** Representative images of H&E and Ki67 staining as well as the expression level of ZDHHC21 as exhibited by IHC assay (magnification 400×, Scale bar, 20 μm). **I**, **J** Percentages of Ki67-positive cells in the indicated tissue are shown (each bar represents the mean ± SD derived from three independent experiments, one-way ANOVA followed by Dunnett’s multiple comparison test or two-tailed Student’s t test, *p < 0.05, ** *p* < 0.01).

The strong relationship between downregulated ZDHHC21 and poor prognosis in DLBCL prompted us to investigate whether ZDHHC21 suppresses DLBCL cell proliferation. To this end, we constructed stable DLBCL cell lines in which ZDHHC21-WT and ZDHHC21-mutant were overexpressed (Supplementary Fig. 3A, cysteine at 120 was mutated to alanine, and palmitoyltransferase activity was lost) in SU-DHL-6 cells, as well as in SU-DHL-2 and Toledo cell lines silenced for ZDHHC21 (Supplementary Fig. 3B). When these SU-DHL-6 cells were tested for their proliferation ability with CCK8 and EdU assays, we found that ectopic overexpression of ZDHHC21-WT, but not ZDHHC21-mutant, significantly decreased the proliferation capabilities (Fig. 2A, Supplementary Fig. 3C, D). Conversely, depletion of ZDHHC21 markedly increased cell proliferation capabilities in SU-DHL-2 and Toledo cells compared with that of the vector cells (Fig. 2B, C, Supplementary Fig. 3C, E).

### ZDHHC21 inhibits DLBCL tumor growth in vivo

Furthermore, the biological function of ZDHHC21 was examined in vivo by subcutaneously inoculating the indicated cells into nude mice. As shown in Fig. 2D, the growth rate of ZDHHC21-WT-overexpressing cells was significantly slowed in vivo, which was confirmed by H&E and Ki-67 staining (Fig. 2H, I). In addition, decreased tumor weights were observed in ZDHHC21-WT-overexpressing cells (Fig. 2E). However, no significant phenotypes were observed in ZDHHC21-mutant-overexpressing cells. In contrast, tumor growth was significantly accelerated when ZDHHC21 was depleted in SU-DHL-2 cells (Fig. 2F, G), and the percentages of Ki-67 positive cells were markedly increased compared with those of the vector cells (Fig. 2J). Taken together, our results indicate that ZDHHC21 is a strong suppressor of DLBCL growth.

### ZDHHC21 interacts with FASN and mediates its S-palmitoylation

**Fig. 3:**
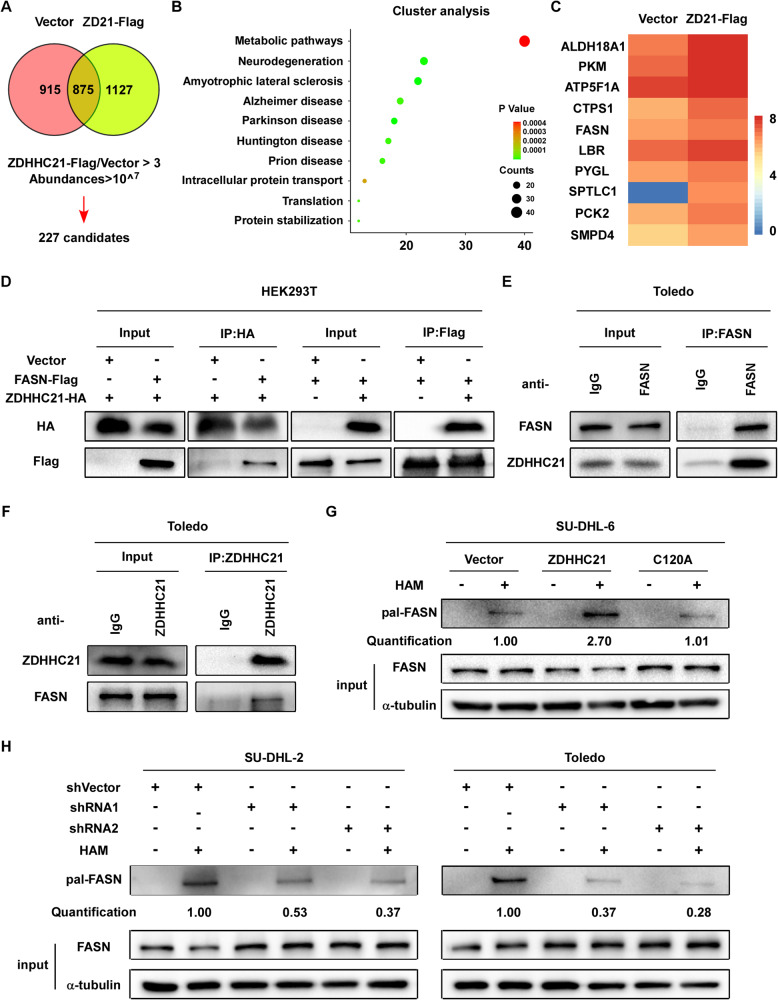
ZDHHC21 interacts with FASN and mediates its S-palmitoylation. **A** Venn diagram showed the enriched proteins of control vector and ZDHHC21-Flag groups. **B** The enriched pathways of ZDHHC21-binding proteins are shown as exhibited by clustering analysis. **C** Heatmap of Log10-peptide abundances change in the control and ZDHHC21-Flag groups showed FASN as a potential binding partner of ZDHHC21. **D** Cell lysates from HEK293T cells expressing Flag-FASN and HA-ZDHHC21 were co-immunopurified with anti-Flag or anti-HA magnetic beads, and western blot assay was performed to validate the interaction between Flag-FASN and HA-ZDHHC21. **E**, **F** Cellular extracts were harvested from Toledo cells and incubated with anti-FASN or anti-ZDHHC21 antibodies. Western blotting assay was performed to confirm the interaction between FASN and ZDHHC21 in DLBCL cells. **G**, **H** The indicated DLBCL cells were lysed in 1 ml lysis buffer and incubated with 10 mM N-ethylmaleimide (NEM) at 4 °C overnight to cap the free cysteine residues. Then, the cell lysates were incubated with 0.5 M hydroxylamine (HAM) for 1 h at room temperature to cleave palmitoylation thioester bonds. Finally, the mixture was incubated with 0.1 mM biotin-HPDP for 1 hour at room temperature and enriched by Streptavidin magnetic beads, followed by SDS-PAGE and immunoblotting. FASN palmitoylation level was quantified by densitometry with Image J.

To further elucidate the mechanism by which ZDHHC21 suppresses DLBCL cell proliferation, co-immunoprecipitation (Co-IP) combined with mass spectrometry was performed to identify the intracellular binding partner of ZDHHC21. As shown in Fig. 3A, 1127 proteins were identified in the ZDHHC21-Flag group, while 915 proteins were identified in the control group. To improve the screening efficiency, we removed proteins whose abundances were less than 10^^7^ and whose ZDHHC21-Flag versus control group fold changes were less than 3. Finally, 227 candidates were identified, a large proportion of which were involved in metabolic pathways, as exhibited by cluster analysis (Fig. 3B). As shown in Fig. 3C, the heatmap showed the top 10 proteins, among which fatty acid synthase attracted our interest because it plays a vital role in fatty acid synthesis which is essential for DLBCL cell growth, and it is not known whether it could be palmitoylated [23, 24]. To validate that ZDHHC21 interacts with FASN, Co-IP using the indicated magnetic beads or antibodies was performed, and the results showed that ZDHHC21 efficiently and reciprocally co-immunoprecipitated with FASN in HEK293T and Toledo cells (Fig. 3D–F). To further investigate whether ZDHHC21 regulates FASN S-palmitoylation, acyl-biotinyl exchange (ABE) assay was performed to quantify the palmitoylation level of FASN. Our data showed that the endogenous palmitoylation level of FASN was dramatically increased in cells ectopically overexpressing ZDHHC21-WT, whereas no similar phenotype was observed in cells overexpressing ZDHHC21-mutant, and palmitoylated FASN was markedly reduced upon ZDHHC21 depletion (Fig. 3G, H). Taken together, our data suggest that ZDHHC21 interactively binds to FASN and mediates its S-palmitoylation.

### ZDHHC21 suppresses FASN expression and fatty acid synthesis

**Fig. 4:**
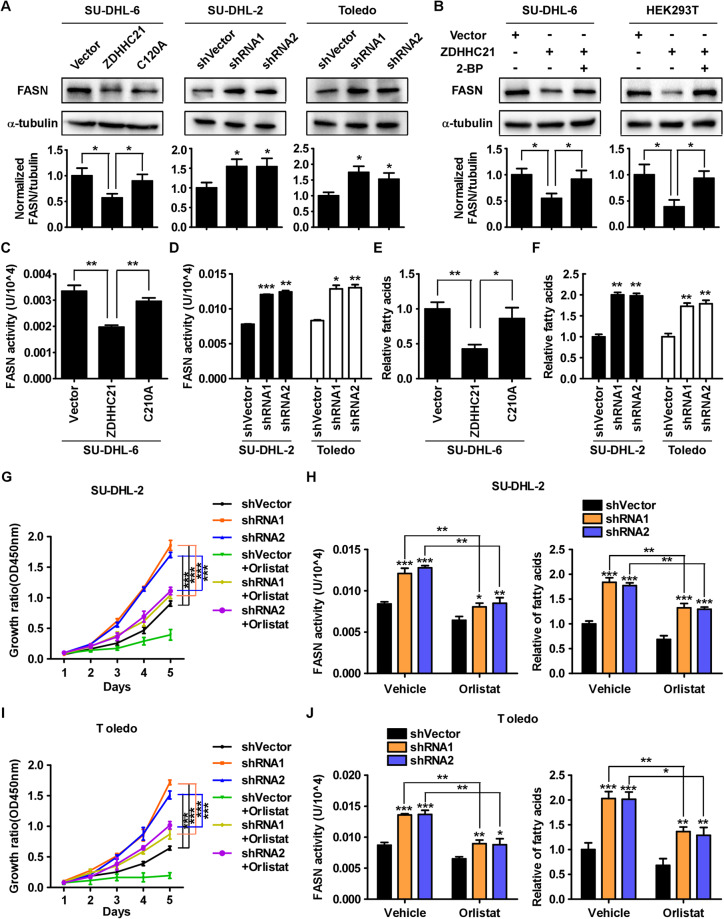
ZDHHC21 suppresses DLBCL proliferation in a FASN-mediated fatty acid synthesis-dependent manner. **A** The expression level of FASN was decreased by overexpression of ZDHHC21-WT, but not the mutant, and increased in ZDHHC21-silenced cells as exhibited by western blot (top). The intensity of FASN expression was quantified by densitometry with α-tubulin as a normalizer (bottom). **B** The expression level of FASN was significantly downregulated in ZDHHC21-WT-overexpressing cells but obviously increased upon 2-BP treatment for 9 h as exhibited by western blot (top) and the intensity of FASN expression was quantified by densitometry with α-tubulin as a normalizer (bottom). **C**, **D** The FASN activity was examined in the indicated cells (each bar represents the mean ± SD derived from three independent experiments, one-way ANOVA followed by Dunnett’s multiple comparison test, **p < 0.01, ***p < 0.001). **E**, **F** The fatty acid level was examined in the indicated cells (each bar represents the mean ± SD derived from three independent experiments, one-way ANOVA followed by Dunnett’s multiple comparison test, *p < 0.05, **p < 0.01). **G**–**J** The control-vector and ZDHHC21-silenced cells were treated with orlistat. The growth rate, FASN activity and the fatty acid level was examined in the indicated cells (each bar represents the mean ± SD derived from three independent experiments, one-way ANOVA followed by Tukey’s multiple comparison test, *p < 0.05, **p < 0.01, ***p < 0.001).

To further investigate whether ZDHHC21 regulates FASN expression, western blotting assay was used to quantify the expression level of FASN. The results suggested that FASN expression was markedly decreased in cells ectopically overexpressing ZDHHC21-WT but not in those overexpressing ZDHHC21-mutant and that significantly elevated FASN expression was observed in ZDHHC21-depleted cells (Fig. 4A). To further confirm that ZDHHC21 regulates FASN expression in a manner dependent on palmitoyltransferase activity, ZDHHC21-overexpressing cells were treated with 2-BP, a general palmitoylation inhibitor, and subsequently, the expression level of FASN was dramatically increased in SU-DHL-6 and HEK293T cells (Fig. 4B). Consistently, FASN enzyme activity and fatty acid level were downregulated in cells overexpressing ZDHHC21-WT but not in those overexpressing ZDHHC21-mutant. Nevertheless, silencing ZDHHC21 significantly enhanced FASN enzyme activity and fatty acid level (Fig. 4C–F). Taken together, our data suggest that ZDHHC21 decreases FASN expression, FASN enzyme activity and fatty acid synthesis in a palmitoylation-dependent manner.

### ZDHHC21 suppresses DLBCL proliferation in a FASN-mediated fatty acid synthesis-dependent manner

As it has been demonstrated that FASN-mediated fatty acid synthesis promotes the proliferation of DLBCL, we next sought to investigate whether ZDHHC21 suppresses DLBCL proliferation in a FASN-mediated fatty acid synthesis-dependent manner. For this purpose, we treated ZDHHC21-depleted DLBCL cells with orlistat, an inhibitor of FASN and found that ZDHHC21 depletion induced increase in proliferation ability, FASN enzyme activity and fatty acid level in DLBCL cells could be attenuated by treatment of orlistat (Fig. 4G–J).

### ZDHHC21 mediates FASN S-palmitoylation at Cys1317 and reduces its protein stability

**Fig. 5:**
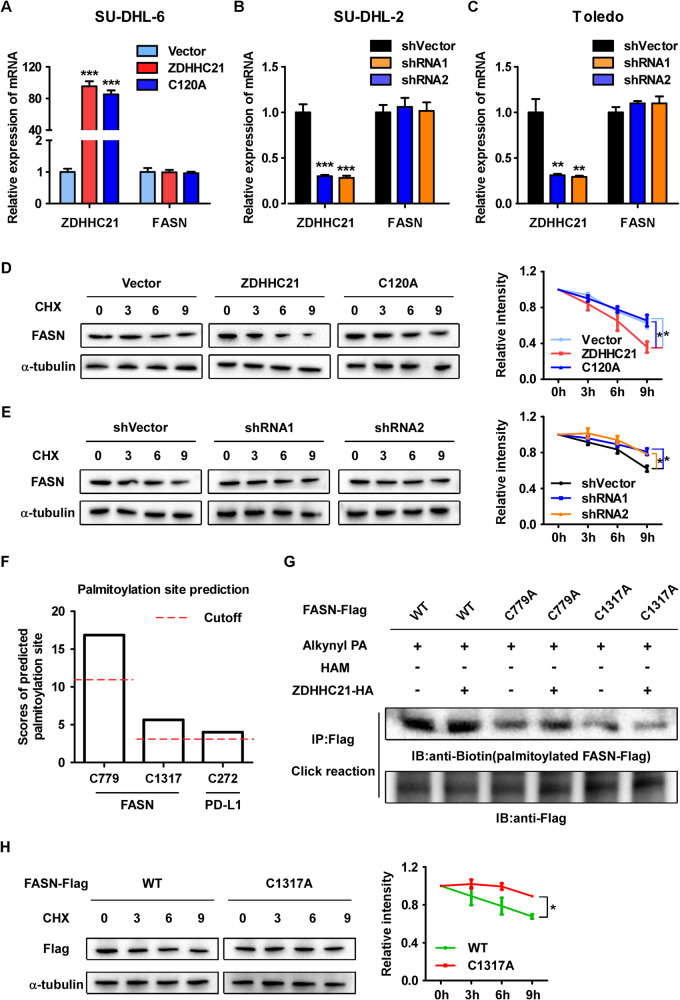
ZDHHC21 mediates FASN S-palmitoylation at Cys1317 and reduces its protein stability. **A**–**C** Validation of ZDHHC21 and FASN mRNA expression level as determined by RT-PCR analyses (each bar represents the mean ± SD derived from three independent experiments, one-way ANOVA followed by Dunnett’s multiple comparison test, ** *p* < 0.01, ***p < 0.001). **D**, **E** The indicated cells were treated with cycloheximide for 0, 3, 6 and 9 h and the expression level of FASN was determined by western blot assay (left), and the intensity of FASN expression for each time point was quantified by densitometry with α-tubulin as a normalizer (right). **F** The potential sites of FASN S-palmitoylation were predicted using the CSS-Palm web server with PD-L1 as a positive control. **G** HEK293T cells were transfected with Flag-tagged wild-type FASN or C779A and C1317A mutants, combined with or without HA-tagged ZDHHC21. After 48 h, the indicated cells were metabolically labeled with alkynyl PA (50 μM) for 12 h. The cell lysate was co-immunopurified with anti-Flag magnetic beads, and then the Flag-FASN palmitoylation level was examined with a click reaction assay. **H** HEK293T cells were transfected with Flag-tagged wild-type FASN or C1317A mutants. Forty-eight hours after transfection, the indicated cells were treated with cycloheximide. Then, western blot assay was performed to examine flag expression (left), and the intensity of flag expression for each time point was quantified by densitometry with α-tubulin as a normalizer (right).

To further investigate the molecular mechanism by which ZDHHC21 regulates FASN expression, RT-PCR assay was performed, and no significant change in FASN mRNA level was observed (Fig. 5A–C). Furthermore, a cycloheximide chase experiment was determined to explore whether ZDHHC21 could regulate FASN protein stability. Our data showed that wild-type ZDHHC21, but not mutant ZDHHC21, decreased the half-life of FASN protein (Fig. 5D), whereas ZDHHC21 depletion significantly prolonged FASN protein stability (Fig. 5E).

To determine the specific site of FASN S-palmitoylation, we first analyzed the FASN sequence and found 46 cysteine residues. Next, we predicted the potential site of FASN S-palmitoylation using the CSS-Palm web server (csspalm.biocuckoo.org/), and the results indicated that Cys779 and Cys1317 displayed potent potential to be palmitoylated. For this analysis, PD-L1, whose S-palmitoylation site has been confirmed, was used as a positive control (Fig. 5F) [15, 25]. Therefore, FASN Cys779 and Cys1317 were mutated to alanine respectively, and alkynyl palmitic acid incorporation assay was performed to examine their S-palmitoylation level. Our data demonstrated that both the C779A and C1317A mutations dramatically reduced the palmitoylation level of FASN. Moreover, ZDHHC21 significantly enhanced the palmitoylation level of wild-type and C779A-mutant FASN but not C1317A-mutant FASN, suggesting that both Cys779 and Cys1317 could be palmitoylated but only palmitoylation at Cys1317 was mediated by ZDHHC21 (Fig. 5G). Further cycloheximide chase experiments also confirmed that the C1317A mutation was related to clearly prolonged FASN half-life compared with that of wild-type FASN (Fig. 5H). Taken together, these data suggest that ZDHHC21 mediates FASN palmitoylation at Cys1317 and decreases its protein stability.

### Targeting ZDHHC21/FASN axis with lanatoside C displays potential antitumor activity in DLBCL

**Fig. 6:**
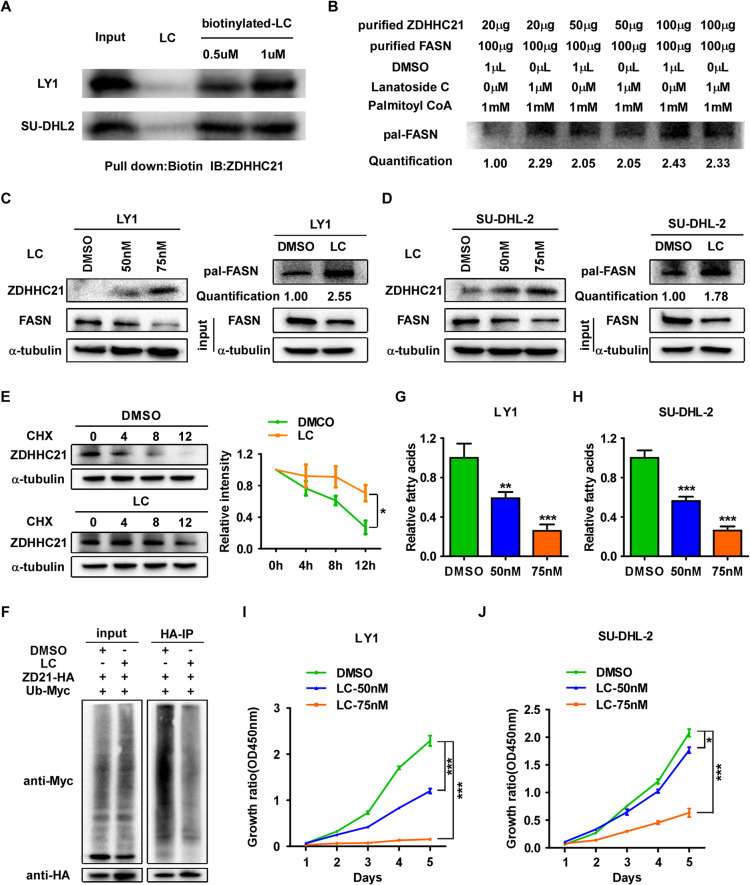
Targeting ZDHHC21/FASN axis with lanatodide C suppresses DLBCL cell proliferation in vitro. **A** Pull down and immunoblotting experiment using streptavidin magnetic beads showed that biotinylated LC interacted with ZDHHC21. **B** 20 μg, 50 μg or 100 μg purified ZDHHC21 with or without lanatoside C (1 μM) was added to a 96-well plate containing reaction buffer (50 mM Tris HCl, 150 mM NaCl, pH 7.4) to incubate for 10 min at 37 °C. Whereafter, 100 μg purified FASN and palmitoyl-CoA (1 mM) were added to incubate for 30 min at 37 °C and FASN palmitoylation level was assessed using ABE assay. Quantification was performed by densitometry. **C**, **D** WB and ABE experiments were performed to examine the ZDHHC21 expression, FASN expression and FASN palmitoylation after treatment with LC for 72 h in LY1 (**C**) and SU-DHL-2 (**D**) cells. **E** DLBCL cells were treated with cycloheximide for 0, 4, 8 and 12 h combined with or without LC treatment. Next, ZDHHC21 expression was determined by western blot assay (left) and the intensity of ZDHHC21 expression for each time point was quantified by densitometry with α-tubulin as a normalizer (right). **F** The ubiquitination assay confirmed that LC largely abrogated ZDHHC21 ubiquitination. **G**, **H** The fatty acid level of the indicated cells is shown after 72 h of lanatodide C treatment (each bar represents the mean ± SD derived from three independent experiments, one-way ANOVA followed by Dunnett’s multiple comparison test, **p < 0.01, ***p < 0.001). **I**, **J** CCK8 assay was performed to determine the growth rate of the indicated cells (each bar represents the mean ± SD derived from three independent experiments, one-way ANOVA followed by Dunnett’s multiple comparison test, *p < 0.05, ***p < 0.001).

The above results suggested that ZDHHC21 significantly suppressed the proliferation of DLBCL, so we reasoned that enhancing ZDHHC21 expression level or its palmitoyltransferase activity could be an effective strategy to attenuate the rapid proliferation of DLBCL cells. For this purpose, we searched the RCSB PDB database (http://www.rcsb.org/) and found a dimensional structure of the human ZDHHC21 protein, which was shown in Supplementary Fig. 4A. On the basis of the RCSB PDB database Model Confidence, the predicted local distance difference test (pLDDT) global scores were 88.56, indicating that the dimensional structure of ZDHHC21 was credible. According to the UniProt database (http://www.uniprot.org/), amino acids 90–140 were defined as the catalytic domain of ZDHHC21, suggesting that this structure could be crucial for ZDHHC21 palmitoyl acyltransferase activity and function. To screen small-molecule compounds that target ZDHHC21, molecular docking was performed between ZDHHC21 (amino acids 90–140) and a small-molecule compound library of FDA-approved drugs, which contains 2,950 compounds. The top-10 ranked candidate small-molecule compounds, based on druggable criteria and binding affinity, were chosen for further analysis. Whereafter, IC50 assay was performed to screen the potential anti-DLBCL drugs, and among the 10 candidates, lanatoside C (LC) appealed to us because of its low IC50 in DLBCL cells and high affinity for ZDHHC21, as exhibited by molecular docking results (Supplementary Fig. 4B, C). LC has previously shown great potential antitumor activity in some solid tumors and multiple myeloma [26, 27]. To further verify the results of molecular docking, LC or biotinylated LC was incubated with LY1 and SU-DHL-2 cell lysates, followed by pull-down assay using streptavidin magnetic beads and immunoblotting experiment. The results showed that ZDHHC21 was detected in the biotinylated LC enriched protein but not in the LC control sample, suggesting that LC interacted with ZDHHC21 (Fig. 6A, Supplementary Fig. 4D). Given that LC was predicted to bind with the catalytic domain of ZDHHC21 according to the results of molecular docking, in vitro enzyme activity assay was performed to confirm whether LC affects the enzymatic function of ZDHHC21. As shown in Fig. 6B, ZDHHC21 enhanced FASN palmitoylation level in a dose-dependent manner. Furthermore, LC could promote FASN palmitoylation in ZDHHC21-20μg group, suggesting that LC might contribute to the enzymatic function of ZDHH21. However, no significant change was observed in ZDHHC21-50μg and ZDHHC21-100μg group. We suspected that LC might contribute to the interaction between ZDHHC21 and FASN or palmitoyl-CoA and the effect would not appear when ZDHHC21 itself was sufficient for FASN complete palmitoylation. To further investigate the effects LC exerted in DLBCL cells, WB and ABE experiments were performed. Our data showed that LC promoted ZDHHC21 expression in a dose-dependent manner, and consistently, FANS palmitoylation was evidently enhanced and FASN expression level was obviously abrogated after treatment with LC in SU-DHL-2 and LY1 cells (Fig. 6C, D). In addition, LC significantly prolonged ZDHHC21 protein half-life and evidently abrogated ZDHHC21 ubiquitination, suggesting that LC repressed ZDHHC21 degradation by the ubiquitin-proteasome pathway (Fig. 6E, F).

To further test this hypothesis, CCK8 and free fatty acid assays were performed, and the results revealed that treatment with LC markedly reduced free fatty acid level and dramatically alleviated the proliferation of LY1 and SU-DHL-2 cells in a dose-dependent manner (Fig. 6G–J).

**Fig. 7:**
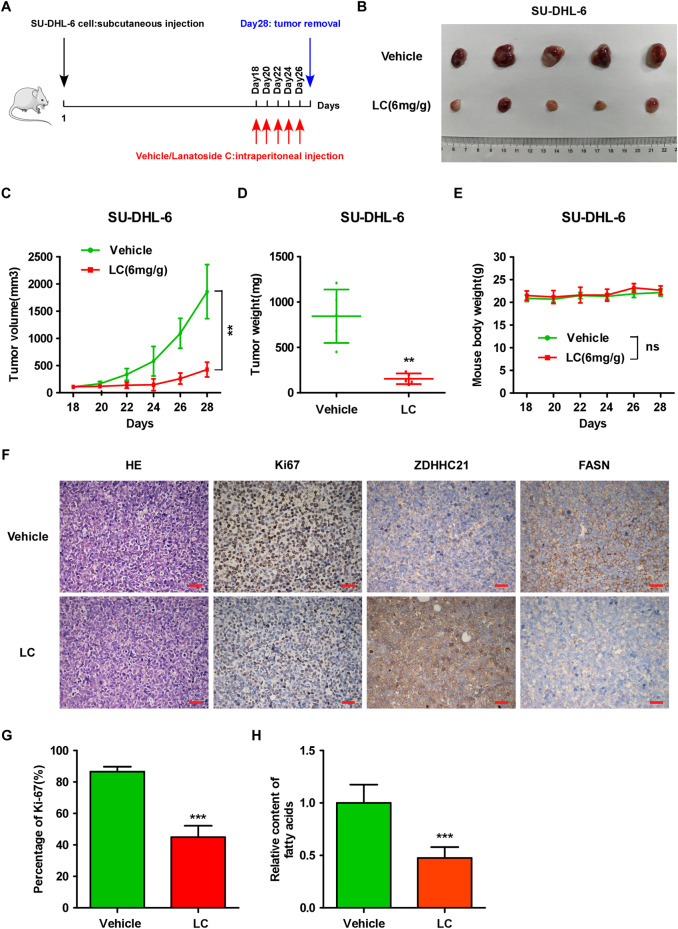
Lanatodide C inhibits DLBCL tumor growth in vivo. **A** A schema showing the experimental design of our mouse experiments. SU-DHL-6 cells were subcutaneously inoculated into nude mice, followed by intraperitoneal administration of vehicle or LC (6 mg/kg) once every two days from day 18 to day 28. At day 28, the xenograft tumors were removed. **B** Representative images of xenograft tumors at the end of the experiments. **C** Tumor growth curve (each bar represents the mean ± SD derived from five independent experiments, two-tailed Student’s t test, **p < 0.01). **D** Tumor weight at day 28 (each bar represents the mean ± SD derived from five independent experiments, two-tailed Student’s t test, ** *p* < 0.01). **E** Body weight of mouse (each bar represents the mean ± SD derived from five independent experiments, ns, not significant). **F** Representative images of H&E and Ki67 staining as well as the expression level of ZDHHC21 and FASN as exhibited by IHC assay (magnification ×400, scale bar, 20 μm). **G** The percentages of Ki67 positive cells are shown (each bar represents the mean ± SD derived from three independent experiments, two-tailed Student’s t test, ***p < 0.001). **H** The fatty acid level of the indicated tissue is shown (each bar represents the mean ± SD derived from three independent experiments, two-tailed Student’s t test, ***p < 0.001).

To verify the antitumor effect of LC in vivo, SU-DHL-6 cells were subcutaneously inoculated into nude mice. When tumors reached almost 100 mm^3^, we randomly divided the mice into 2 groups, followed by intraperitoneal administration of vehicle or LC (6 mg/kg) every two days (Fig. 7A). As shown in Fig. 7B–E, the growth rate of DLBCL cells and tumor weights dramatically declined upon treatment with LC, but no significant change was observed in mice body weights compared with those of the vehicle control group. Consistently, markedly upregulated ZDHHC21 and significantly reduced FASN expression level and percentage of Ki67-positive cells were observed in the LC group, as exhibited by IHC assay (Fig. 7F, G). In addition, the tumor tissues derived from the vehicle control and LC groups were harvested, and the fatty acid content was measured. Our data showed that fatty acid level was significantly decreased after treatment with LC in vivo (Fig. 7H). Taken together, the above results collectively determined that targeting ZDHHC21/FASN axis with LC exerts powerful antitumor activity in DLBCL in vitro and in vivo.

## Discussion

A previous study has shown that ZDHHC21 mediates endogenous androgen receptor palmitoylation and enhances membrane trafficking in breast cancer cells [18]. In addition, a recent study reported that ZDHHC21 is highly expressed in acute myeloid leukemia cells and mediates AK2 palmitoylation as well as mitochondrial localization to promote cancer development [19]. Our study demonstrates that palmitoyltransferase ZDHHC21 is downregulated in DLBCL cells and suppresses their proliferation. These findings indicate that ZDHHC21 acts as a tumor suppressor to inhibit malignant progression of DLBCL by facilitating FASN palmitoylation, suggesting that aberrant expression of ZDHHC21 differs in different types of cancer and that the specific biological function of ZDHHC21 may be context-dependent.

Increased de novo fatty acid synthesis is an important hallmark of cancer cells, and fatty acid biosynthesis is catalyzed by multifunctional, homodimeric fatty acid synthase [23, 28, 29]. Moreover, DLBCL cells are highly reliant on lipids for cell proliferation [4–7]. Therefore, it is of great importance to explore the therapeutic vulnerability of de novo fatty acid synthesis in DLBCL cells. However, the molecular mechanisms underlying the significantly enhanced fatty acid synthesis in DLBCL are not completely understood. Our study suggested that ZDHHC21 suppressed FASN expression and fatty acid synthesis in a palmitoylation-dependent manner in DLBCL. Notably, an FDA-approved compound LC was identified and found to increase ZDHHC21 expression and decrease FASN expression as well as fatty acid synthesis, consequently suppressing DLBCL growth in vitro and in vivo. Previous studies have shown that LC inhibits the proliferation of various solid tumors and multiple myeloma [26, 27, 30, 31]. Our study is the first demonstration that LC exhibits anti-tumor activity in lymphoma by targeting palmitoylation and fatty acid synthesis, suggesting that ZDHHC21/FASN axis can potentially be a promising therapeutic target for patients with DLBCL.

Elevated FASN expression is frequently observed in multiple cancers and has been demonstrated to promote cancer cell proliferation, survival and metastasis, which results in a higher risk of disease-related death [23, 24, 32–34]. The expression level of FASN in cancer cells can be regulated at various biological levels. For instance, SREBP is one of the most crucial transcription factors that contributes to the elevated FASN level in cancer cells [35, 36]. In addition, various post-translational modifications, including ubiquitination, acetylation and sumoylation, have emerged as important contributors to FASN upregulation in multiple cancers [37–39]. A previous study has suggested that the ubiquitin-specific protease USP2a interacts with and removes ubiquitin from FASN, subsequently preventing ubiquitin-mediated degradation in prostate cancer [40]. To our knowledge, our study represents the first demonstration that ZDHHC21-mediated FASN S-palmitoylation at Cys1317 inhibits FASN protein stability, exhibiting a novel mode of post-translational regulation of FASN expression and extending our understanding of FASN regulation in cancer cells.

Protein S-palmitoylation is a post-translational modification that goes hand in hand with fatty acid synthesis and fatty acid synthetase [10, 41]. Previous studies have suggested that FASN synthesizes endogenous fatty acid that can be converted into palmitoyl-CoA and then bind to cysteine residues of the target proteins and numerous proteins were reported to be palmitoylated in a FASN-dependent manner. For example, Ali et al. demonstrated that FASN mediates EGFR palmitoylation in EGFR-mutated non-small cell lung cancer cells and regulates the sensitivity of tyrosine kinase inhibitors [42]. In addition, MYD88 was found to be FASN-dependently palmitoylated by ZDHHC6 in neutrophils [43]. However, whether FASN itself can be palmitoylated remains unknown. Our present study found that FASN could be palmitoylated by ZDHHC21 at Cys1317 in DLBCL, leading to a decline in its protein stability. Interestingly, ZDHHC21-mediated palmitoylation-dependent downregulation of FASN results in decreased fatty acid synthesis, which may in turn reduce palmitoylation of other FASN-dependent palmitoylated proteins because of decreased palmitate level; therefore, these results suggest that global protein palmitoylation regulation mediated by ZDHHC21 could exist in this context in DLBCL.

### Supplementary information

Supplementary Figures

Supplementary Table 1

Supplementary Table 2

## Data Availability

The data supporting the findings of this study are available from the corresponding author upon reasonable request.
