# Supplementary material for: Targeting ZDHHC21/FASN axis for the treatment of diffuse large B-cell lymphoma
Source: Leukemia. 2024 Jan 9;38(2):351–64. doi: 10.1038/s41375-023-02130-5 (PMC10844076; doi:10.1038/s41375-023-02130-5)
Supplement: Supplementary file 1 — Supplementary Figures [file 41375_2023_2130_MOESM1_ESM.docx]

**Supplemental Figures**

**
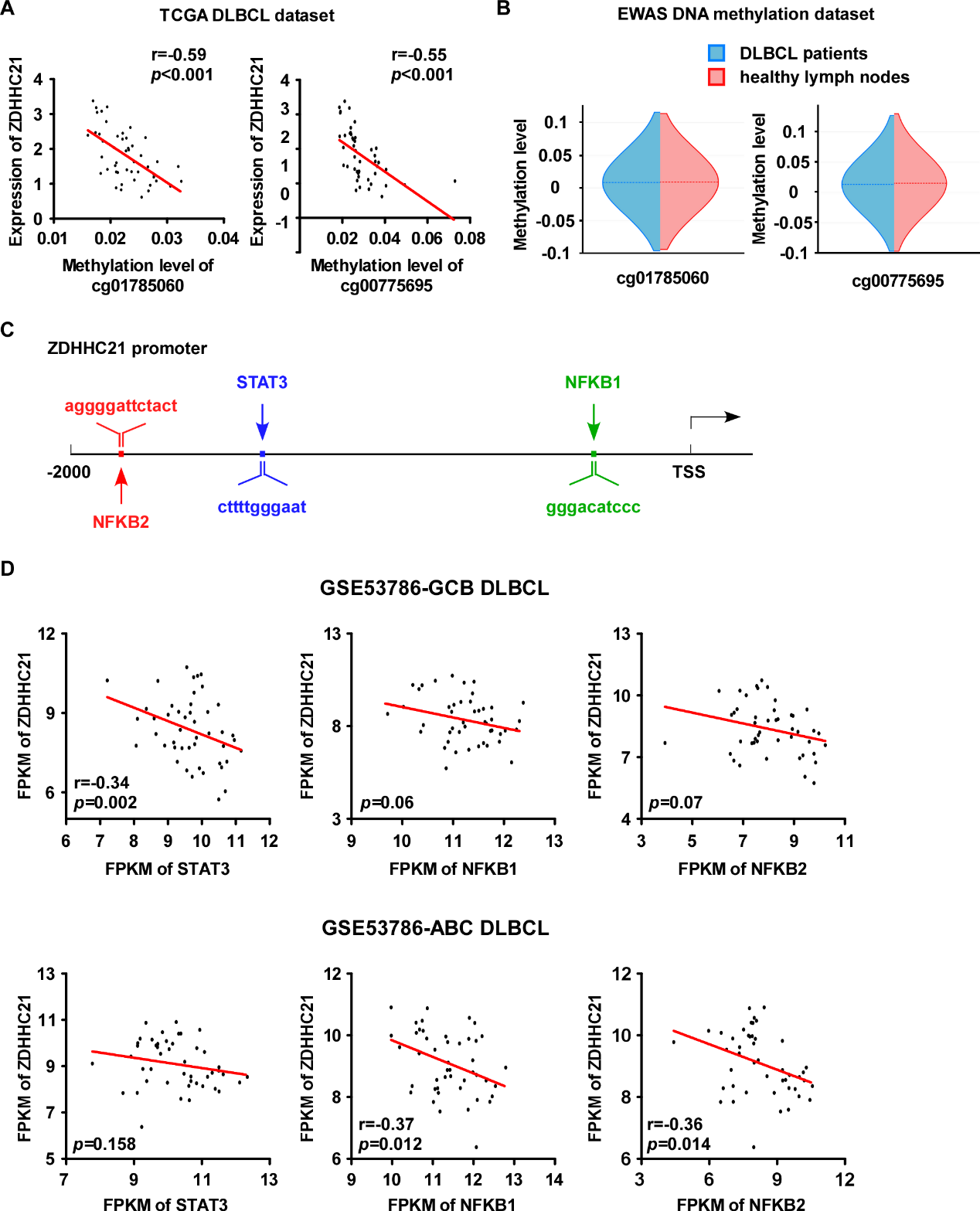
**

**Supplemental Fig 1. The circumstances of low ZDHHC21 expression in DLBCL.** (A) The correlation between methylation level of probe cg01785060 as well as cg00775695 and ZDHHC21 expression level in TCGA DLBCL dataset. (B) The methylation level of probe cg01785060 and cg00775695 in DLBCL patients and healthy lymph node control in EWAS DNA methylation dataset. (C) Predicted binding sites of NFKB1, NFKB2 and STAT3 in ZDHHC21 promoter analyzed by JASPAR dataset. (D) The correlation between NFKB1, NFKB2 as well as STAT3 expression and ZDHHC21 expression in GCB and ABC DLBCL patients from GSE53786 dataset.


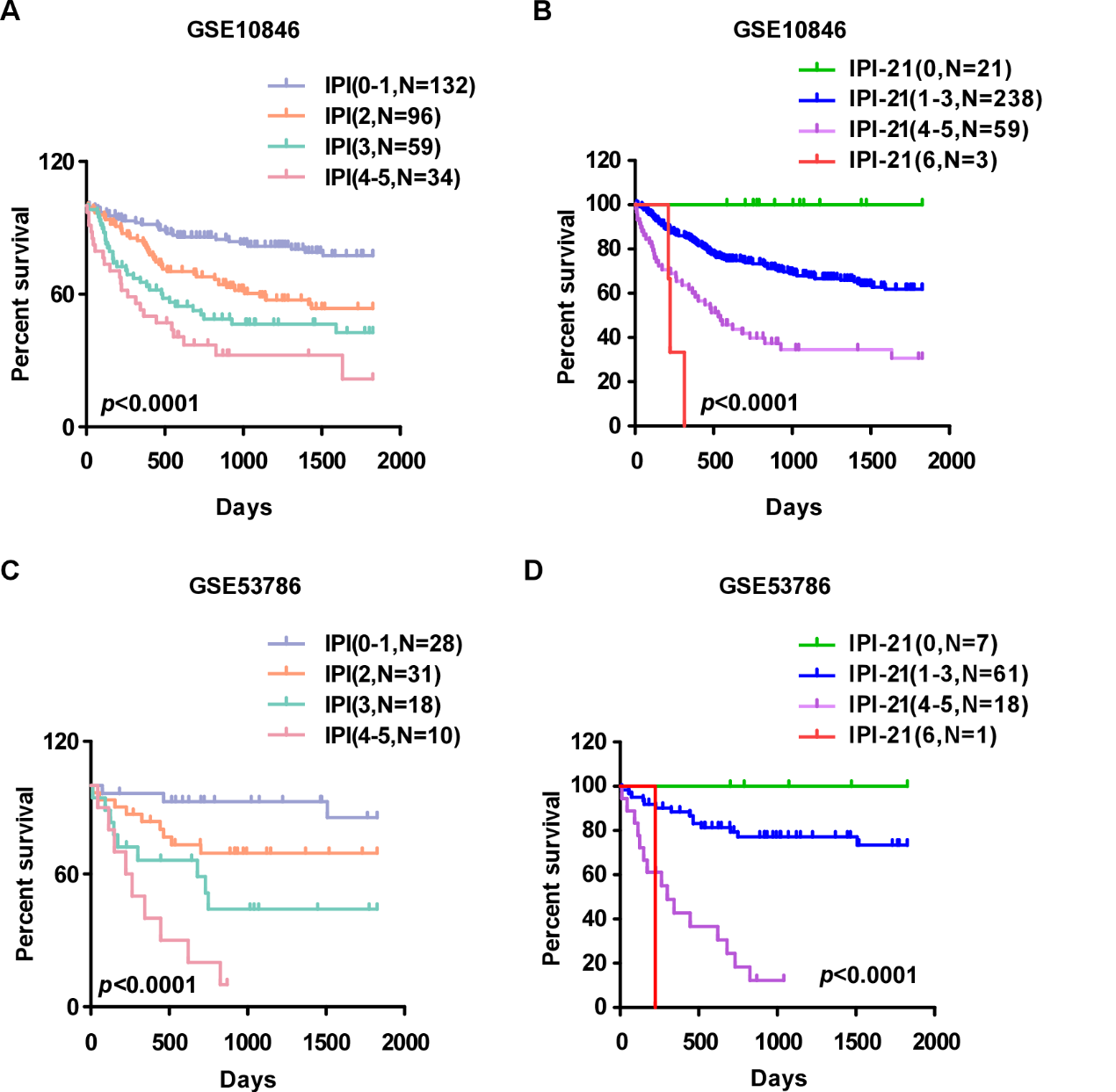


**Supplemental Fig 2. ZDHHC21 expression is downregulated in DLBCL with prognostic value.** (A) Kaplan-Meier survival analysis of overall survival in the GSE10846 DLBCL cohort based on International Prognostic Index (IPI) scores. (B) We combined IPI scores with ZDHHC21 expression (with ZDHHC21 low expression defined as 1 point) and established the IPI-21 system. Kaplan-Meier survival analysis of overall survival in GSE10846 DLBCL cohorts based on IPI-21 scores. (C-D) Overall survival in GSE53786 DLBCL cohorts was determined by Kaplan-Meier analysis based on IPI scores (C) and IPI-21 scores (D).


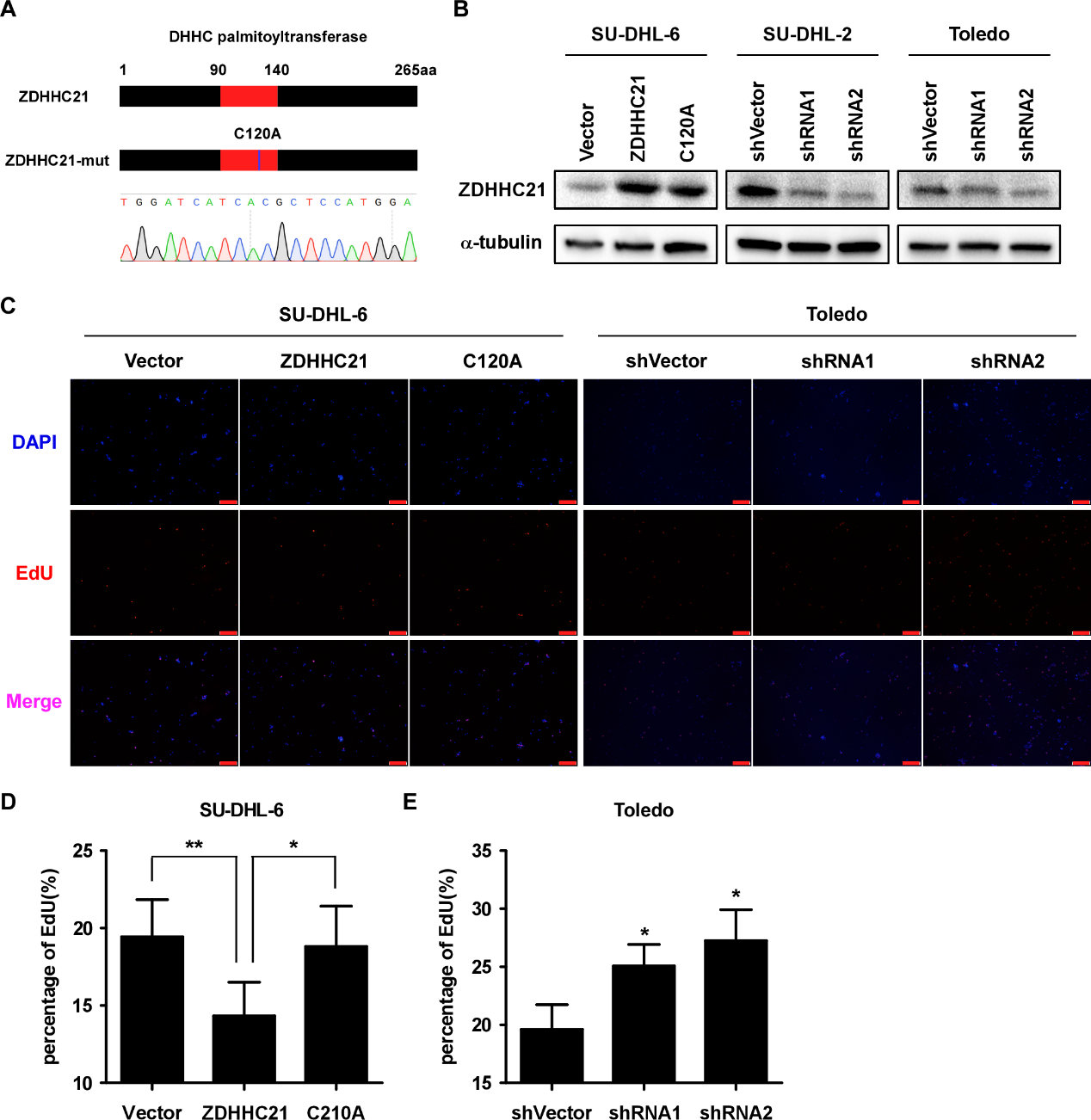


**Supplemental Fig 3. ZDHHC21 suppresses DLBCL cell proliferation.** (A) ZDHHC21 DNA sequencing, prediction of ZDHHC21 palmitoyltransferase regions and mutation of the active site, as shown with a blue box. (B) Western blot analyses of ZDHHC21 and ZDHHC21-C120A protein expression levels. (C-E) The EdU assay was performed and representative images (C, magnification 100×, scale bar, 100 μm) as well as percentages of EdU-positive cells (D-E) are shown (each bar represents the mean ± SD derived from three independent experiments, one-way ANOVA followed by Dunnett’s multiple comparison test, **p* < 0.05, ** *p* < 0.01).


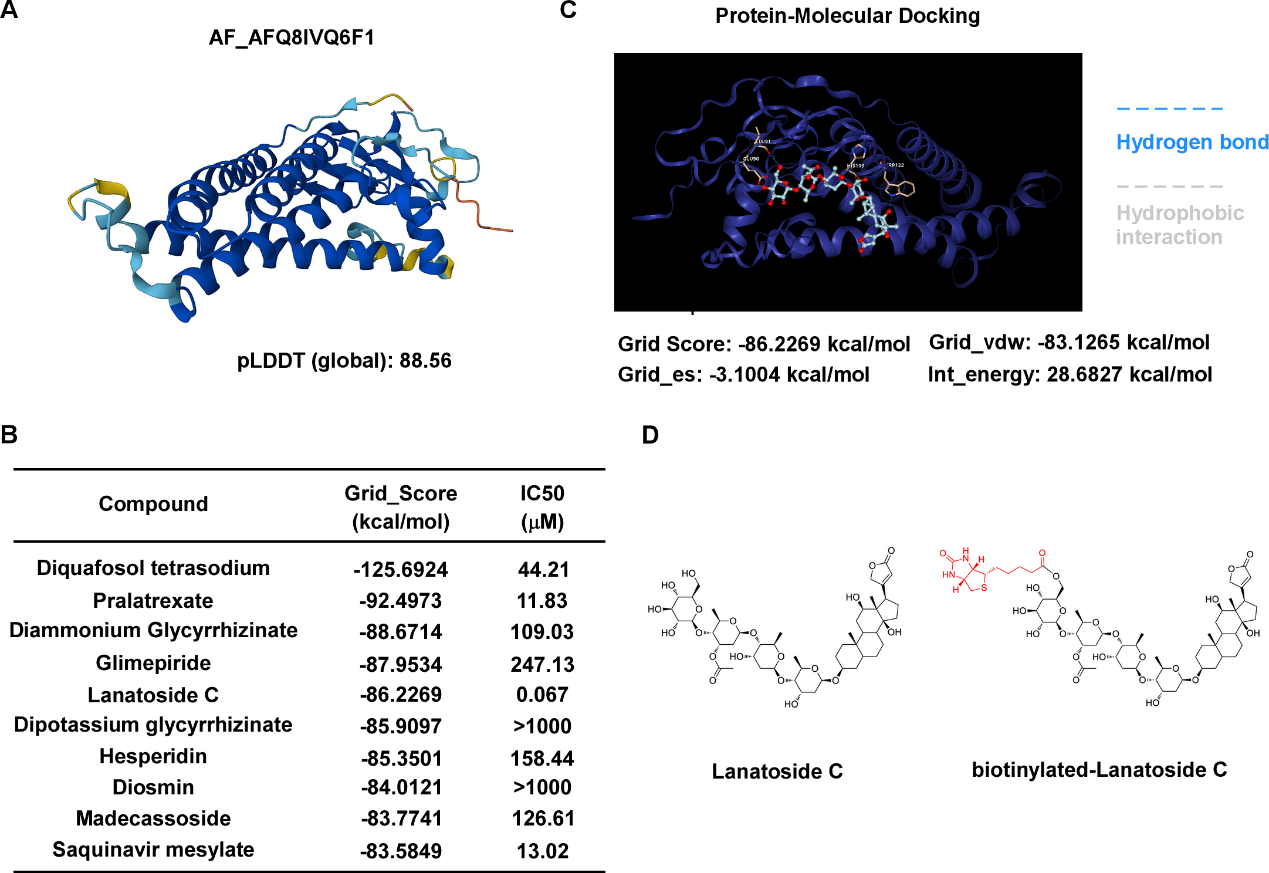


**Supplemental Fig 4. Screening of anti-DLBCL drugs as exhibited by molecular docking.** (A) Computed 3D structure model of human palmitoyltransferase ZDHHC21 and model confidence scores of ZDHHC21 by the RCSB PDB database. (B) The Grid_score of candidate compounds with ZDHHC21 and IC50 of candidates in DLBCL cells. (C) A model of LC binding with the ZDHHC21 protein generated by molecular docking. (D) Chemical structures of LC and biotinylated LC.
